# Overwhelmed by Learning in Lockdown: Effects of Covid-19-enforced Homeschooling on Parents’ Wellbeing

**DOI:** 10.1007/s11205-022-02936-3

**Published:** 2022-06-23

**Authors:** Marieke Heers, Oliver Lipps

**Affiliations:** 1 FORS, c/o University of Lausanne, Lausanne, Switzerland; 2 University of Bern, Bern, Switzerland

**Keywords:** Covid-19, Educational policy, Homeschooling, Parental involvement, Public health, Social inequalities, Wellbeing

## Abstract

With the closure of schools due to the Covid-19-pandemic, parents of schoolchildren had to quickly adapt their daily schedules by taking over responsibilities of homeschooling for their children, while arranging their own schedule. This study first identifies the parents who are most challenged by having to ensure homeschooling responsibilities and then assesses how homeschooling affects different dimensions of parents’ wellbeing. Analyzing data from a large general population-representative panel survey in Switzerland, we compare subjective wellbeing before the Covid-19-outbreak to wellbeing at the end of the semi-lockdown. Almost one fifth of parents report being sometimes overwhelmed by homeschooling obligations for their children. Women, mid-aged and lower-educated individuals as well as those with young children and a lower income are particularly overwhelmed. Being sometimes overwhelmed by homeschooling does not cause changes in life satisfaction, stress and negative affect. Yet, it leads to a decrease in positive affect. We derive recommendations for educational and public health policies.

## Introduction

The Covid-19-pandemic has forced families to quickly adapt their everyday routines (Coyne et al., 2020; Falkingham, Evandrou, Qin, & Vlachantoni, 2020). They had to practice social distancing, potentially experienced reduced workloads implying reduced household income, had to deal the fear of infection and many families suddenly found themselves in a small, restricted place during the entire day (Altena et al., 2020; Brooks et al., 2020; Fegert et al., 2020). As a major challenge many parents suddenly had to monitor their children’s learning efforts at home (Doyle, 2020; Parczewska, 2020; Reimer et al., 2021; Spinelli et al., 2020). These conditions represent a potential threat to parents’ wellbeing. At the same time, the restrictions and reduction in work-related and social obligations meant that families could spend more time together and stressful factors from everyday life were relieved. This may explain the results from previous research showing that stress levels decreased during the first weeks during which the measures were active (Kuhn et al., 2021).

Not all parents perceive providing homeschooling in the same way (Brom et al., 2020; Settersten et al., 2020). While some parents feel at ease with intervening in their children’s education, others have more difficulty and are unconfident about their competencies (Bol, 2020; Parczewska, 2020). In this article, homeschooling refers to mandatory home education, or home learning, due to the school closures as a measure taken during the Covid-19-pandemic. In the context of the Covid-19-pandemic, studies also refer to distance learning (e.g. Champeaux, Mangiavacchi, Marchetta, & Piccoli, 2020). So far, it is unclear if the obligation to provide homeschooling impacts parents’ wellbeing in a positive or in a negative way, therefore, more knowledge thereon is needed. The aim of our study is twofold. First, we compare parents who felt at ease with homeschooling to those who were overwhelmed by it. Second, we analyze the effects of being overwhelmed by homeschooling on different dimensions of parents’ subjective wellbeing. To this end, we use panel data from a population-representative Swiss sample comparing parents’ subjective wellbeing during the Covid-19-period to that a few months before.

The questions raised in this article are highly policy relevant. First, better knowing the profiles of parents who need support with homeschooling is crucial for educational policymakers and practitioners; homeschooling will probably remain part of education during the pandemic and might become more widespread afterwards. Second, to prevent long-term consequences of the pandemic on mental health and wellbeing (OECD, 2020; Thorell et al., 2021; World Health Organization, 2020), shedding more light on the consequences of providing homeschooling on parents’ wellbeing is highly relevant from a general societal and public policy perspective (Diener et al., 2013; López Ulloa et al., 2013; Maccagnan et al., 2019). Third, parents’ mental health is an important predictor of children’s cognitive and socioemotional development (Fontanesi et al., 2020; Lau et al., 2018; Reupert & Maybery, 2016). Fourth, the results from this study are useful beyond the Covid-19-pandemic by relating parents’ perceived efficiency in intervening in their children’s education to their own wellbeing; an area in which only little is known.

## Background

This study links three research strands. First, the literature on parental involvement in their children’s education. Second, that on the characteristics of homeschooling, with a particular focus on the Covid-19-pandemic. Third, the recent literature on how the Covid-19-pandemic relates to subjective wellbeing. The evidence from the literature will guide the empirical analyses of this study.

### Parental Educational Involvement and Homeschooling

Parents differ in terms of how they are involved in the education of their children. Some parents are actively involved while others rather refrain from it. In particular, parents with a higher socio-economic status (SES) generally value education more and encourage their children to participate in educational activities (Esping-Andersen, 2008; Lareau, 2011). These parents tend to be more involved in their children’s education and have more knowledge about successfully navigating their children through the educational system (Chin & Phillips, 2004; Esping-Andersen, 2008; Fitzmaurice et al., 2020; Lareau, 2011; McNeal, 1999; Roksa & Potter, 2011). Compared to lower-SES parents, they can more easily activate their economic, cultural and social capital and align their practices closely with those of the school (Fitzmaurice et al., 2020). Some studies report that lower-SES parents tend to be as much involved as higher-class parents; however, their involvement is less effective in terms of children’s learning outcomes (Cabus & Ariës, 2017). With respect to providing homeschooling during the Covid-19-pandemic, this suggests that for lower-SES parents it is more difficult to replicate schooling at home and that providing homeschooling is more challenging for them.

Studies on homeschooling prior to the Covid-19-pandemic provide important insights: across families, large differences in the educational principles that homeschooling is based on reflect different parental strategies, educational approaches and worldviews (Guterman & Neuman, 2018; Tilhou, 2020). Accordingly, homeschooling activities range on a continuum of many hours of pre-planned structured activities to no structured activities at all (Guterman & Neuman, 2018). Traditionally, homeschooling was a choice made by parents (Guterman & Neuman, 2018; Neuman & Aviram, 2003; Ray, 2017). Only recently, amongst others due to digitalization, homeschooling has become more widespread across Western countries and more accessible for larger parts of the population (Guterman & Neuman, 2018; Jolly & Matthews, 2020; Ray, 2017; Tilhou, 2020). With the outbreak of the Covid-19-pandemic, homeschooling was no longer a choice but imposed on parents. Hence, the pandemic can be considered an exogenous event that allows verifying the existing evidence on homeschooling based on a sample, which is representative for the total population.

Studies that have started to analyze homeschooling in the context of the Covid-19-pandemic point out a number of factors that affect the success of its implementation. These factors include parental educational and literacy levels, poverty status, physical and psychological health, characteristics of the living environment (e.g. number of rooms per person) as well as the availability of computers and internet access (Doyle, 2020; Fisher et al., 2020). Time constraints represent another barrier to homeschooling. Moreover, research on Covid-19-related homeschooling shows that children of parents with lower educational backgrounds have worse learning conditions at home (Huebener & Schmitz, 2020) and they risk to be negatively affected from school closures in terms of educational achievement (Eyles, Gibbons, & Montebruno Bondi, 2020). Moreover, parents’ perception of their capability to help their children with schoolwork is higher amongst those who have a higher education degree themselves (Bol, 2020). Prior studies also suggest that needs for parental support with homework as well as support for homeschooling decrease with increasing child-age (Collins et al., 2021; Eccles & Harold, 1993; Hoover-Dempsey et al., 2001). Another crucial insight from this literature is that there are large heterogeneities in learning environments at home (Andrew et al., 2020), which are likely to widen social inequalities (Bol, 2020; Fisher et al., 2020; Reimer et al., 2021).

The literature suggests that mothers are more involved in homeschooling activities than fathers. Research on telework has revealed that mothers who work from home more frequently engage more in enrichment activities, such as playing and reading, with their children. This tendency is not observed for fathers (Kim, 2020). Similarly, compared to fathers, mothers tend to carry out more homeschooling at the expense of paid work (Collins et al., 2021; Guterman & Neuman, 2018; Zhou, Hertog, Kolpashnikova, & Kan, 2020). How mothers and fathers differ in the extent to which they are overwhelmed by having to provide homeschooling and how this affects their wellbeing has not yet been established.

### Homeschooling, Covid-19 and Parents’ Subjective Wellbeing

Subjective wellbeing refers to individuals’ evaluations of their lives, including judgements of life satisfaction and evaluations of feelings (e.g. moods and emotions) (Diener & Chan, 2011). Covid-19 and the measures taken by national and local policymakers are likely to have detrimental effects on individual wellbeing (Falkingham et al., 2020; OECD, 2020), such as increased daytime stress, anxiety and depression levels (Altena et al., 2020; Burton-Jeangros et al., 2020; Horesh & Brown, 2020; World Health Organization, 2020).

Previous research on quarantine has shown that social isolation can lead to mental health problems, including depression, low mood, irritability, insomnia, anger and emotional exhaustion (Brooks et al., 2020; Fegert et al., 2020). While some studies reported reductions in wellbeing due to the pandemic (Zhou et al., 2020), the evidence is not univocal. A study on the French population before and after the outbreak of the Covid-19-pandemic found that wellbeing as well as the perception of health had improved during the lockdown compared to previous years. Yet, there are differences across social groups: the most financially vulnerable or individuals living in smaller homes report reductions in wellbeing (Recchi et al., 2020).

Working parents are likely to experience a decrease in their subjective wellbeing, as they were challenged by the requirements from work while having to monitor their children’s homeschooling (Coyne et al., 2020; Falkingham et al., 2020; Fontanesi et al., 2020; Griffith, 2020). A study from Germany, which has examined how school and day care center closures affected parents’ wellbeing, found a reduction in wellbeing for individuals with young children, women, and individuals with lower secondary schooling qualifications (Huebener et al., 2021). In the UK, parents with childcare responsibilities reported lower levels of wellbeing during the pandemic compared to those without such responsibilities (Etheridge & Spantig, 2020). Other studies, however, did not find evidence for reduced wellbeing as a result of additional childcare responsibilities (Adams-Prassl, Boneva, Golin, & Rauh, 2020).

While most of the anticipated effects of the pandemic on wellbeing are negative, the literature also refers to potential benefits (Fegert et al., 2020). Most families could spend more time together. Being forced to stay at home can bring rest and relaxation into family life. External stressors, such as commuting, disappear and might lead to a strengthened cohesion between family members (Fegert et al., 2020).

Whether being overwhelmed by having to provide homeschooling affects parents’ wellbeing has not yet been assessed. Here, we assess the possible relationships between being overwhelmed by homeschooling and four wellbeing indicators: Life satisfaction, stress, positive and negative affect. Stress is particularly relevant regarding vulnerable societal groups (Pearlin, 1999), who we also expect to be particularly overwhelmed by homeschooling. Life satisfaction and positive and negative affect are complementary components of subjective wellbeing (Diener et al., 1999; Galambos et al., 2020). As elaborated in the following sections, these wellbeing indicators seem particularly relevant in relation to homeschooling.

#### Homeschooling and Life Satisfaction

Parents, who suddenly have to adjust their work schedules and other life domains and, in addition, have to provide homeschooling to their children, are likely to experience a decrease in life satisfaction. Life satisfaction refers to a cognitive judgmental process, in which individuals compare themselves with a standard set for themselves individually (Diener et al., 1985). Life satisfaction is one of the most commonly analyzed wellbeing indicators (Diener et al., 1999).

A study in Switzerland found that, across the population, life satisfaction did not change during the lockdown. However, more vulnerable groups, i.e., individuals who are socially isolated, who have relatively few socioeconomic resources, or an increased workload, reported a decrease in life satisfaction (Kuhn et al., 2021). For Germany, Huebener et al. (2021) found a decline in life satisfaction for individuals with young children. Based on panel data for Germany, Entringer et al. (2020) found no significant change in life satisfaction compared to previous years. There is no clear evidence on the effect of homeschooling obligations on individual-level life satisfaction.

#### Homeschooling and Stress

Several studies suggest that the conditions of the pandemic and particularly the burden of having to provide homeschooling cause a growth in perceived stress (Fegert et al., 2020; Spinelli et al., 2020; Thorell et al., 2021). A study on stress during the lockdown in Switzerland has revealed a strong increase in stress in the general population; yet, a fourth of the population has reported a better mental health status compared to the time prior to the lockdown (Burton-Jeangros et al., 2020). That study measured mental health during the lockdown and when the measures were partially lifted. At both measurements, respondents were asked to compare their current health status to that prior to the Covid-19-outbreak. The data collection took place within a short timeframe and was based on a convenience sample (de Quervain et al., 2020).

Having to provide homeschooling while not feeling prepared and equipped for it is likely to cause frustration amongst parents (Parczewska, 2020). Research from Italy has shown that parents, who experience taking care of their children’s education as more difficult, are more stressed (Spinelli et al., 2020). In France, lockdown-related stress has not changed significantly across different time points during the pandemic, i.e., in April and May 2020. Yet, higher levels of stress are reported by individuals spending long working days at home (Recchi et al., 2020). If a similar mechanism is at play for homeschooling, we should observe that parents who are overwhelmed by it experience higher levels of stress.

None of the above-mentioned studies measured stress-levels prior to the pandemic. A study on Switzerland that has measured pre-pandemic stress did not find a change in mean stress. Yet, stress levels decreased among high earners, workers on short-time work and highly educated individuals (Kuhn et al., 2021). The role of being overwhelmed by having to provide homeschooling has not been investigated.

#### Homeschooling and Positive and Negative Affect

The affective wellbeing dimensions include the positive and the negative affect (Diener, 1984; Diener et al., 1985), which are composed of either positive or negative moods, emotions or affects (Scherer, Wranik, Sangsue, Tran, & Scherer, 2004; Watson et al., 1988). The positive affect includes feelings like strength, energy, optimism and joy. The negative affect includes feelings of anxiety and depression, anger, sadness, worry and desperation.

Confinement, loss of usual routine, and reduced social contacts are likely to cause boredom, frustration, anxiety and a sense of isolation, which distresses individuals (Brooks et al., 2020; World Health Organization, 2020). Findings based on panel data for Germany show that the pandemic has led to increased symptoms of depression and anxiety compared to 2019; yet, the levels are comparable to those measured in 2016. Lades et al. (2020) have assessed within-person variance in emotional wellbeing during the pandemic and showed that levels of negative affect were notably higher for individuals who provide homeschooling for their children. However, the authors did not measure negative affect prior to the pandemic. A study from Poland on parents’ role in providing education to their children reports negative emotions such as anger, annoyance, irritation, fear and helplessness (Parczewska, 2020).

Against this background, we test if parents, who feel overwhelmed by having to provide homeschooling, are more likely to report lower levels of positive and higher levels of negative affect, compared to parents who are at ease with providing homeschooling.

#### Homeschooling, Wellbeing and Social Inequalities

Prior studies have also shown that women’s wellbeing is more negatively affected by the pandemic than men’s (Huebener et al., 2021), e.g. in terms of mental health (Daly, Sutin, & Robinson, 2020) and sleep loss (Falkingham et al., 2020). Moreover, gendered allocations of childcare, i.e., mothers providing the majority of care and education for children (Falkingham et al., 2020; Settersten et al., 2020), make it likely that women’s wellbeing is more strongly affected by having to provide homeschooling than men’s. With respect to the educational background the evidence is mixed; some studies report that individuals with lower educational backgrounds are more affected in terms of reductions of wellbeing (Huebener et al., 2021), while others report that a higher educational degree relates to a larger increase in mental health problems (Daly et al., 2020).

## Context, Data and Method

### Context

This study focuses on Switzerland, which compared to many other countries was less strongly hit by the first wave of the pandemic (Roser, Ritchie, Ortiz-Ospina, & Hasell, 2020) and had implemented moderate lockdown measures. Although authorities recommended individuals to stay at home, outdoor activities were allowed as long as less than six individuals gathered and physical distancing was assured (Federal Office of Public Health (FOPH), 2020b). Schools were closed from March 8 and started reopening on May 11, depending on educational level and canton (Federal Office of Public Health (FOPH), 2020a; Refle et al., 2020). During this period, education took place digitally and from home. Children of parents working in essential jobs were provided with childcare and educational institutions remained accessible for them. This refers to approximately 8% of households with children below 18.^1^ On the 11th of May, primary and secondary schools started reopening and teaching at upper secondary schools and institutions resumed for groups of up to five pupils. Teaching at upper secondary, vocational, and higher education resumed on June 6 (Federal Office of Public Health (FOPH), 2020a, 2020b; Refle et al., 2020). The Swiss language-regions differed in the extent to which they were affected by the first wave of the Covid-19-pandemic. The Italian-speaking region and the French-speaking cantons of Geneva and Vaud were most affected. There was heterogeneity in terms of measures taken across cantons, with stronger measures implemented in the more affected regions. Nevertheless, on average, during the lockdown in Switzerland, the level of wellbeing and satisfaction with life remained on the same level as prior to the Covid-19-pandemic (Ehrler, Monsch, & Steinmetz, 2020; Kuhn et al., 2021).

### Data and Sample

We use data from the Swiss Household Panel (SHP), an annual panel survey, which started in 1999 using a probability-based sample of the Swiss population living in private households (SHP Group, 2020; Tillmann et al., 2016). During the Covid-19 crisis, the SHP conducted a between-wave SHP Covid-19 Study among respondents of the previous panel wave 21 (for details see Refle et al., 2020). 8772 persons aged 14–99 from 5,540 households received an invitation to complete a web-based questionnaire. Nonrespondents to the web questionnaire received a reminder that included a paper version of the questionnaire. With 5843 responding individuals, the response rate was 67%. The *SHP Covid-19 Study* was conducted between May 12 and June 26, 2020. While schools in Switzerland were closed between March 8 and May 11, 2020 (Federal Office of Public Health (FOPH), 2020a), the questionnaire asked specifically to refer to periods when schools were closed. Respondents of the SHP Covid-19 Study were matched with data from wave 21 that was conducted between September 2019 and March 2020 (95% was completed before December 17, 2019), referred to as the *pre-Covid-19-wave*.^2^ With an average of six months, the period between the two waves of data collection was rather short. Therefore, we expect little change in respondents’ living conditions except the outbreak of the pandemic.

Compared to the 2019/2020 wave, there is little selectivity in the data collected by the SHP *Covid-19 Study*. Specifically, wellbeing and social participation were not significantly related to response propensity (Kuhn et al., 2021). We use weights to produce population-representative results. Our analysis sample includes all adult individuals with children (until age 17) and consists of 842 individuals.

The questions on wellbeing in the Covid-19 questionnaire were formulated with the same wording as the ones in the previous waves. This is crucial to calculate valid change scores. A potential concern with the timing of the survey is that we cannot be certain that respondents relate the questions on wellbeing to the period when schools were closed. This is because some interviews took place when schools had already started to reopen. Moreover, in the literature on wellbeing, there is a discussion on measuring wellbeing retrospectively (Kahn & Juster, 2002). In the wellbeing questions, there was no explicit reference to the time when schools were closed (also because the wellbeing questions are asked to respondents without school children as well). However, from the framing of the Covid-19 questionnaire it is clear that all questions refer to the time of the strictest measures including school closures. Nevertheless, this timing issue might lead to biased results and is therefore taken into account in the analysis below.

### Measures

We consider four indicators of subjective wellbeing (Refle et al., 2020; Voorpostel et al., 2020). All four are measured in the *Covid-19 Study* as well as in the *pre-Covid-19-wave*: *Life satisfaction* is measured by the question “In general, how satisfied are you with your life?” (0 = not at all satisfied, …, 10 = completely satisfied). *Stress* is measured by asking “How often have you felt stressed during the last four weeks?” (1 = never, …, 5 = very often). *Positive affect* was measured as follows: “Are you often plenty of strength, energy and optimism, if 0 means “never” and 10 “always”?” The question on *negative affect* was formulated “Do you often have negative feelings such as having the blues, being desperate, suffering from anxiety or depression, if 0 means “never” and 10 “always”?” (Scherer et al., 2004).

The variable of interest is *being sometimes overwhelmed by having to provide homeschooling* and was measured in the *Covid-19 Study* with the question “Helping my child/children keep up with schoolwork overwhelms me sometimes” (1 = disagree completely, 2 = disagree somewhat, 3 = partly agree, partly disagree, 4 = agree somewhat, 5 = agree completely).

As mentioned above, some respondents answered the survey after the schools had re-opened again, and we cannot be sure if these respondents relate the “overwhelmed”-question to the time during school closures and if they recall their experience correctly. We therefore define a variable *homeschooling* that indicates if homeschooling was effective at the moment of the interview: the variable homeschooling takes the value 1 if the interview took place before the 11th of May 2020 for families with children up to the age of 14 and before the 6th of June for families with children aged 15 to 17. It takes the value 0 if homeschooling is no longer effective at the time of the interview.

The following independent variables, measured in 2019/2020, are included from the *pre-Covid-19-SHP-data-wave*: respondents’ sex (0 = female; 1 = male), age and age squared, tertiary educational attainment (0 = no; 1 = yes includes vocational high schools and university education)^3^ and an indicator if at least one child younger than 12 is living in the household. Assuming that practical and emotional social support plays an important role (Fegert et al., 2020), we control for the presence of a cohabiting partner. We also account for households’ equivalent income,^4^ which proxies factors such as space and learning equipment. We further include the language region (German, French, Italian). As prior studies have identified, the fear of infection as a potential stressor (Brooks et al., 2020), based on the *Covid-19 Study* we control for whether the respondent is in the Covid-19-risk group. The latter indicator is based on the question “Do you consider yourself part of the population at risk of developing complications from a Covid-19 infection, for example based on your age or pre-existing health conditions?”^5^ A few independent variables needed to be imputed (chained equations).

Table 1 presents descriptive statistics for our analysis sample. While 38% of the respondents in the *Covid-19-wave* are not at all overwhelmed by having to provide homeschooling to their children, almost a fifth (18%) is sometimes somewhat or completely overwhelmed. In order to compare individuals who are sometimes overwhelmed to those who are not, we calculate a binary indicator: 0 = not overwhelmed, 1 = sometimes overwhelmed. Columns 2 and 3 in Table 1 show the descriptive statistics along these two categories.

**Table 1:** Sample characteristics (proportions), full sample and by being sometimes overwhelmed vs. not being overwhelmed

	(1)	(2)	(3)
	Full sample	Not overwhelmed	Sometimes overwhelmed
*Overwhelmed by homeschooling*			
Disagree completely	38.1		
Disagree somewhat	26.8		
Partly agree, partly disagree	17.2		
Agree somewhat	12.6		
Agree completely	5.2		
Man	43.9	46.1	34.0
Woman	56.1	53.9	66.0
Age (mean)	45.6	45.7	45.0
*Tertiary education*			
No	54.3	52.6	62.0
Yes	45.7	47.4	38.0
Youngest child 12 or older	33.1	34.7	26.0
Youngest child younger 12	66.7	65.3	74.0
No cohabiting partner	9.7	9.5	10.7
Cohabiting partner	90.3	90.5	89.3
Not Covid-19-risk group	88.7	88.3	90.7
Covid-19-risk group	11.3	11.7	9.3
Equivalent income (in 10,000 Sfr.)	6.1	6.2	5.5
*Language region*			
German-speaking	64.6	68.8	45.3
French-speaking	29.5	25.1	49.3
Italian-speaking	5.9	6.1	5.3
Interview while homeschooling	9.4	9.8	7.3
N	842	692	150
%	100		

SHP Covid-19 Study 2020, unweighted (N = 842)

While 56% of the sample members are women, their proportion represents 66% amongst those who are sometimes overwhelmed by homeschooling. The bivariate relationship between gender and being overwhelmed is significant, according to the Spearman’s rho statistic (Prob >|t|< 0.05). On average, respondents are 46 years old and 46% have a tertiary educational attainment. 67% have a child younger than 12 living in the household; this age group can be expected to require more parental resources with respect to homeschooling. 90% have a cohabitating partner. 11% are in the Covid-19-risk group. Household equivalent income is lower amongst individuals who are sometimes overwhelmed by having to provide homeschooling. While 65% of the sample are from the German-speaking part of Switzerland, amongst those who are sometimes overwhelmed by homeschooling this proportion amounts to 45%, while 49% of those who are overwhelmed are from the French-speaking region. This relationship is significant. Almost 10% of the interviews took place while homeschooling was still effective.

### Method

The analysis proceeds as follows: first, we regress the variable overwhelmed by homeschooling on the socio-demographic factors described above. Specifically, we run an ordered logistic regression with the five categories of being overwhelmed as the dependent variable.

Second, for each of the four wellbeing indicators, we compare the means for the full sample and across the two levels of being overwhelmed by having to provide homeschooling. For each wellbeing dimension, we consider three components: the outcome measured a few months before the outbreak of the pandemic, the outcome measured during the pandemic and the change score, i.e., the intra-individual difference in the outcome between the Covid-19- and the pre-Covid-19-wave. Change score models reduce omitted variable bias (Morgan & Winship, 2015). We estimate change score models and regress the change scores on being sometimes overwhelmed by having to provide homeschooling, the timing of the interview and the socio-demographic characteristics presented in Table 1.

## Results

### Who is Overwhelmed by Having to Provide Homeschooling?

In order to unfold the relationship between being overwhelmed by homeschooling and different indicators of parental wellbeing, we first analyze who reported to be overwhelmed. Beta coefficients from the ordered logistic regressions are presented in Table 2.^6^ Women are more overwhelmed than men. Age is an important predictor of being overwhelmed: given the concave relationship between overwhelmed and age, parents just above 50 experience the highest levels of being overwhelmed. This inverse U-shaped relationship between age and being overwhelmed corresponds to what is observed in parts of the literature on the relationship between age and subjective wellbeing, which, however, is also very much contested (Blanchflower & Oswald, 2008; Brüderl & Ludwig, 2015; Easterlin, 2006; Galambos et al., 2020; López Ulloa et al., 2013). Compared to parents with a tertiary educational attainment, those with a lower educational attainment are more often sometimes overwhelmed by having to provide homeschooling. This is in line with research showing that higher educated individuals have more resources to draw from in order to support their children’s education (Esping-Andersen, 2008; Lareau, 2011). Parents of children younger than 12 are more overwhelmed than parents of older children. This aligns with studies showing that older children have more self-guided homeschooling approaches than younger children (Collins et al., 2021). Neither having a cohabiting partner nor being in the Covid-19-risk group is related to higher levels of being overwhelmed. Income is negatively related to the extent of being overwhelmed. Finally, compared to Swiss-German speakers the French-speakers are more overwhelmed by having to provide homeschooling, the Italian speakers less. The first may be because the French-speaking part of Switzerland was more severely hit by the pandemic. However, it may also be related to cultural differences and to heterogeneities in school systems.^7^

**Table 2:** Results from ordered logistic regression predicting being sometimes overwhelmed by having to provide homeschooling (beta coefficients)

Overwhelmed	Coef.
Woman	0.45***
Age	0.30***
Age^2^	− 0.003***
*Tertiary education*	− 0.61***
Child < 12	0.79***
Cohabiting partner	− 0.09
Covid-19-risk group	0.04
Equivalent income/10′000	− 0.06***
*Linguistic region*	
German-speaking	(ref.)
French-speaking	0.94***
Italian-speaking	− 0.59*
Pseudo R^2^	0.05

Swiss Household Panel, wave 2019 and, SHP Covid-19 wave, weighted, N = 842

*** *p* < 0.001, ** *p* < 0.01, * *p* < 0.05

A robustness check with a model controlling for additional factors confirms the results presented in Table 2. These factors include: working at home, employment status (differentiating employed, self-employed, unemployed, inactive), number of persons living in the household, foreigners from non-neighboring countries, number of schoolchildren^8^ in the household, a changed work-life balance^9^ and whether, in 2019, respondents participated in a web- or telephone survey. Being a foreigner and a decreased work-life balance are related to higher levels of being overwhelmed.

### Wellbeing Before and During the Lockdown and its Relationship with Being Overwhelmed by Homeschooling

Table 3 presents the indicators of wellbeing from the pre-Covid-19- and the Covid-19-wave as well as changes in the wellbeing indicators (i.e., the change scores, CS). The indicators do not reveal a uniform picture. For those individuals who are sometimes overwhelmed, we find a decrease in life satisfaction, stress and positive affect, and an increase in negative affect. For those who are not overwhelmed, the change score is only significant for stress and negative affect. If we only consider the sample that participated in the interview while homeschooling was still effective, the results are similar (see Table 6 in the Appendix). These observations suggest that the pandemic has nuanced effects on individuals’ wellbeing. In the following sections, we use multivariate models to assess to what extent sometimes being overwhelmed vs. not being overwhelmed relate to changes in the wellbeing indicators.

**Table 3:** Descriptive statistics (means and standard errors) of wellbeing indicators, pre-Covid-19 and Covid-19-wave and change scores (CS) for full sample and by being sometimes overwhelmed

Wellbeing indicator	Pre-Covid-19	Covid-19	CS	N
Life satisfaction (1–10)	7.9 (0.04)	7.9 (0.05)	− 0.08 (0.04)	842
Not overwhelmed	8.0 (0.04)	7.9 (0.05)	− 0.04 (0.05)	692
Sometimes overwhelmed	7.7 (0.11)	7.5 (0.12)	− 0.25 (0.12)	150
Stress (1–5)	3.1 (0.04)	2.8 (0.03)	− 0.32 (0.04)	842
Not overwhelmed	3.0 (0.04)	2.7 (0.04)	− 0.35 (0.04)	692
Sometimes overwhelmed	3.3 (0.09)	3.0 (0.09)	− 0.22 (0.09)	150
Positive affect (1–10)	7.1 (0.06)	7.0 (0.06)	− 0.04 (0.06)	842
Not overwhelmed	7.1 (0.06)	7.2 (0.06)	0.04 (0.06)	692
Sometimes overwhelmed	6.9 (0.16)	6.5 (0.18)	− 0.40 (17)	150
Negative affect (1–10)	2.0 (0.07)	2.4 (0.07)	0.41 (0.06)	842
Not overwhelmed	1.8 (0.07)	2.2 (0.07)	0.35 (0.06)	692
Sometimes overwhelmed	2.8 (0.20)	3.4 (0.22)	0.69 (0.19)	150

Swiss Household Panel, wave 2019 and, SHP Covid-19 wave, weighted

**Table 4:** OLS results for the relationship between being sometimes overwhelmed by homeschooling and changes in wellbeing between the pre-Covid-19 and the Covid-19-wave

	Life satisfaction			
	M1	M2	M3	M3 + controls
Homeschooling during interview		− 0.04	− 0.00	− 0.12
*Not overwhelmed (ref.)*				
Sometimes overwhelmed	− 0.21*	− 0.21*	− 0.19*	− 0.10
Homeschooling*Overwhelmed			− 0.28	− 0.31
Constant	− 0.04	− 0.04	− 0.04	− 2.48**

	Stress			
	M1	M2	M3	M3 + controls
Homeschooling during interview		0.03	0.06	0.23
*Not overwhelmed (ref.)*				
Sometimes overwhelmed	0.12	0.13	0.14	0.07
Homeschooling*Overwhelmed			− 0.25	− 0.28
Constant	− 0.35***	− 0.35***	− 0.35***	− 3.37***

	Positive affect			
	M1	M2	M3	M3 + controls
Homeschooling during interview		0.12	0.12	0.18
*Not overwhelmed*(ref.)				
Sometimes overwhelmed	− 0.44***	− 0.44***	− 0.44***	− 0.40**
Homeschooling*Overwhelmed			− 0.05	− 0.06
Constant	0.04	0.03	0.03	− 1.11
R^2^	0.010	0.011	0.011	0.024

	Negative affect			
	M1	M2	M3	M3 + controls
Homeschooling during interview		0.36	0.26	0.10
*Not overwhelmed* (ref.)				
Sometimes overwhelmed	0.34**	0.35**	0.31*	0.13
Homeschooling*Overwhelmed			0.82	0.95
Constant	0.35***	0.32***	0.33***	− 1.88
R^2^	0.005	0.008	0.010	0.049

Swiss Household Panel, wave 2019 and, SHP Covid-19 wave, weighted, N = 842

*** *p* < 0.001, ** *p* < 0.01, * *p* < 0.05. The control variables are sex, age, age^2^, tertiary educational attainment, having a child below 12, having a cohabiting partner, being in the Covid-19-risk group, equivalent income and language region

### Homeschooling, Being Overwhelmed by it and Wellbeing

For each wellbeing indicator, we estimate four models of which the results are presented in Table 4. We first estimate the effect of being sometimes overwhelmed by homeschooling (similar to Table 3; M1), then control for having reported during times of homeschooling (M2). Model M3 adds an interaction of both variables to make sure respondents report being sometimes overwhelmed during the time that homeschooling was still going on. Finally, model M4 controls the level of the wellbeing indicators for the sociodemographic variables listed in Table 2 to check if the effects of being sometimes overwhelmed on changes in wellbeing are mediated by sociodemographic variables.

#### Homeschooling and Life Satisfaction

Life satisfaction has decreased for individuals who are sometimes overwhelmed, but not for those who are not overwhelmed. Timing (homeschooling at the moment of the interview) does not affect the effect of being overwhelmed on a changed life satisfaction. The reduction due to being overwhelmed by homeschooling also does not change when being interviewed during the homeschooling period. However, the socio-demographic control variables moderate the reduction in life satisfaction due to being overwhelmed such that this coefficient becomes insignificant. To conclude, being overwhelmed by homeschooling led to a reduction in life satisfaction after and during times of homeschooling, but this is mostly moderated by the socio-demographic characteristics.

#### Homeschooling and Stress

Sometimes being overwhelmed by providing homeschooling to their children has no effect on a changed stress between the pre-Covid-19- and the Covid-19-wave. Timing does not explain this zero effect. In addition, being overwhelmed while interviewed during the homeschooling period does not change this (zero) effect of being overwhelmed on stress. Finally, also controlling for socio-demographic characteristics does not change the absence of an effect of being overwhelmed on stress change.

#### Homeschooling and Positive Affect

Positive affect has decreased for individuals who are sometimes overwhelmed by homeschooling, but not for those who are not overwhelmed. Controlling for timing does not change the effect of being overwhelmed on a changed positive affect, nor does being overwhelmed by homeschooling while being interviewed during the homeschooling period. In addition, the effect is not moderated by the socio-demographic variables. To conclude, being overwhelmed by homeschooling led to a reduction in positive affect independent of whether homeschooling was effective.

#### Homeschooling and Negative Affect

Next, we consider parents’ reported levels of negative affect. Individuals who are sometimes overwhelmed by homeschooling report higher levels of negative affect, both before and during the pandemic. Accordingly, the change score is higher for individuals who are sometimes overwhelmed compared to those who are not. Timing does not explain the increase in negative affect due to being overwhelmed. Being overwhelmed and interviewed during times of homeschooling does also not change the effect. However, once we control for the socio-demographic variables, the effect of being overwhelmed on negative affect is no longer significant. To conclude, similar to the effect on life satisfaction, being overwhelmed by homeschooling led to an increased negative effect, but this is mostly moderated by the socio-demographic characteristics.

## Conclusion and Discussion

This study has identified the parents who are most challenged by having to provide homeschooling and how this affects different dimensions of their wellbeing; namely, general life satisfaction, stress and positive and negative affect. To that end, we have analyzed panel data allowing us to assess individual-level changes in wellbeing a few months prior to the outbreak of the Covid-19-pandemic to that reported during the lockdown in spring 2020.

While some parents were not at all overwhelmed by having to provide homeschooling, almost a fifth report being sometimes overwhelmed. Some groups are particularly overwhelmed: women and middle-age parents as well as those with low educational attainment, with lower income, and with children younger than 12. Moreover, parents in the French-speaking region of Switzerland, hit more seriously by the first wave of the pandemic, were more often overwhelmed. These groups would benefit from support with homeschooling responsibilities. Particularly, parents with lower educational attainment as well as poorer individuals may perceive not having all the required resources to support their children’s learning, while higher educated or richer parents can more easily activate their economic, cultural and social capital and thereby create a homeschooling environment that aligns their practices closely with those of the school (Bol, 2020; Fitzmaurice et al., 2020).

Comparing wellbeing prior to the Covid-19-pandemic to that after the outbreak, parents report a decrease in stress and an increase in negative affect. Amongst those who are sometimes overwhelmed by having to provide homeschooling there is, in addition, a decrease in life satisfaction and positive affect, and an increase in negative affect.

Multivariate analyses using individual-level change scores show that sometimes being overwhelmed by homeschooling does not *cause* changes in life satisfaction, stress and negative affect. Yet, it *causes* a decrease in positive affect. In addition, our models show that the results are robust against the (late) timing of the interviews, where most interviews were actually conducted after schools already started reopening. Therefore, there seem to be no recall errors. This means that being overwhelmed by homeschooling leads to a reduction in positive affect, independent of whether homeschooling was still effective or not.

Our findings reveal the importance of taking the timing of the interview into account. With respect to the wellbeing measures analyzed here, this is particularly relevant with respect to life satisfaction and negative affect: during times of homeschooling parents’ wellbeing is affected negatively in these dimensions stronger than beyond, even though this is mostly explained by the control variables. This finding could also point to recall errors, which supports doubts on retrospective measures of wellbeing (Kahn & Juster, 2002). If all parents would have been interviewed during the effective homeschooling period, the identified relationships would even be stronger and, therefore, our estimates are rather conservative.

Taken together, being sometimes overwhelmed by having to provide homeschooling causes changes in parents’ wellbeing. If homeschooling is to be (partly) continued, wellbeing might be further impacted. Therefore, future research has to investigate the long-term implications and should also focus on other wellbeing domains, such as sleep problems or satisfaction with the relationship. Future research should also evaluate the support parents need to provide homeschooling. Potential avenues for future research are how teachers can better support parents or if parents would require more flexibility from their employers in order to feel comfortable in providing homeschooling. It should also be noted that as part of the lockdown measures schools were closed abruptly; implying that schools, teachers, parents and children were not prepared for it. This led into very different homeschooling experiences (Champeaux et al., 2020; Parczewska, 2020; Reimer et al., 2021); for example, in terms of teacher involvement but also the quality of the distance learning. Unfortunately, our data do not contain information on how families actually experienced homeschooling, but future research should assess this issue. In the empirical analysis, we have used change scores. While they reduce omitted variable bias (Morgan & Winship, 2015), we cannot rule out that other characteristics may lead to higher levels of feeling overwhelmed by homeschooling and might be related to different changes of wellbeing. Moreover, our study is limited by a relatively low number of cases who were overwhelmed by homeschooling and, in particular, by a low number of respondents who have been interviewed at the same time. Therefore, the results must be interpreted carefully. Future research should assess in more detail how interview timing relates to validity of wellbeing measures and this should be taken into account in survey designs. In empirical analyses, interview timing should be controlled for more rigorously. Finally, research should further explore if positive affect is impacted in the longer run.

Several conclusions regarding education and health policymaking can be drawn from our study: a considerable proportion of parents is overwhelmed by the responsibility to provide homeschooling. Educational policymaking may consider developing strategies to support these parents in creating an effective learning environment at home. For example, programs that support parents in more efficiently intervening in homeschooling and in the education of their children would be valuable. These should be tailored to groups of parents running a high risk of being overwhelmed by homeschooling responsibilities. Moreover, offering additional programs for children from those families is crucial to ensure they do not fall behind. During the first wave of the pandemic, Switzerland left educational institutions somewhat open for children of parents working in essential jobs, a similar offer should be created for children whose parents cannot ensure effective homeschooling.

In this study, we have focused on the population of parents with children below 18, other groups of the population might be affected to a different extent. Finally, while this study has analyzed changes in wellbeing prior to the Covid-19-outbreak to those during the outbreak, the pandemic is not yet over. It will be crucial to see how parents are dealing with the challenges in the longer run. Therefore, future research and particularly panel data should assess the longer-term impacts of having to provide homeschooling on parents’ but also children’s wellbeing. Closely monitoring the situation will be crucial for educational and public health practitioners.

The findings from this study are relevant beyond the period of the Covid-19-pandemic. While already before, homeschooling became increasingly widespread (Guterman & Neuman, 2018; Jolly & Matthews, 2020; Ray, 2017; Tilhou, 2020), the new experience and digital tools might reinforce this trend. Moreover, hybrid learning, i.e. a mix of distance education and learning taking place in schools, might become more common. Our study shows that, in fact, and even under the challenging conditions of the pandemic, in many families homeschooling worked well. This is crucial with respect to educational policies, which, so far, in many countries, did not permit homeschooling on a regular basis. With this study, we shed some light on the conditions for homeschooling to be successful, and who are the parents who might be at ease with providing it. Yet, more research is needed on the conditions for successful homeschooling beyond the Covid-19-pandemic, when the conditions and needs would be completely different.

Finally, our study puts forward that the consequences of homeschooling and distance learning go well beyond questions of educational attainment. In particular, this is the first study to assess the consequences of being overwhelmed by homeschooling on parental wellbeing. Most studies have analyzed the impact on educational inequalities across students (Bol, 2020; Reimer et al., 2021). Thereby, this study opens avenues for new research directions and raises our attention to the fact that the whole family is affected and factors related to families’ wellbeing must be included in the discussions on educational developments.

## Appendix

See Tables 5 and 6.

**Table 5:** Results from ordered logistic regression predicting being overwhelmed by having to provide homeschooling, standardized coefficients for continuous variables (age, age^2^, Equivalent income/10,000)

Overwhelmed	Coef.
Woman	0.45***
Age	2.14***
Age^2^	− 1.96***
*Tertiary education*	− 0.61***
Child < 12	0.79***
Cohabiting partner	− 0.09
Covid-19-risk group	0.04
Equivalent income/10,000	− 0.22***
Linguistic region	
German-speaking	(ref.)
French-speaking	0.94***
Italian-speaking	− 0.59*
Pseudo R^2^	0.05

Swiss Household Panel, wave 2019 and, SHP Covid-19 wave, weighted, N = 842

*** *p* < 0.001, ** *p* < 0.01, * *p* < 0.05

**Table 6:** Descriptive statistics (means and standard errors) of wellbeing indicators, pre-Covid-19 and Covid-19-wave and change scores (CS) by being sometimes overwhelmed for respondents who were providing homeschooling during the time of the interview

Wellbeing indicator	Pre-Covid-19	Covid-19	CS	N
Life satisfaction (1–10)	8.2 (0.13)	8.1 (0.14)	− 0.10 (0.12)	79
Not overwhelmed	8.2 (0.14)	8.1 (0.15)	− 0.04 (0.13)	68
Sometimes overwhelmed	8.9 (0.36)	8.3 (0.37)	− 0.52 (0.34)	11
Stress (1–5)	2.8 (0.13)	2.5 (0.12)	− 0.31 (0.14)	79
Not overwhelmed	2.8 (0.14)	2.5 (0.13)	− 0.29 (0.15)	68
Sometimes overwhelmed	2.9 (0.31)	2.5 (0.34)	− 0.40 (0.50)	11
Positive affect (1–10)	7.2 (0.19)	7.3 (0.20)	0.10 (0.17)	79
Not overwhelmed	7.1 (0.21)	7.2 (0.22)	0.16 (0.18)	68
Sometimes overwhelmed	8.0 (0.31)	7.7 (0.50)	− 0.33 (0.40)	11
Negative affect (1–10)	1.4 (0.19)	2.2 (0.22)	0.72 (0.16)	79
Not overwhelmed	1.5 (0.21)	2.1 (0.24)	0.58 (0.17)	68
Sometimes overwhelmed	1.1 (0.47)	2.8 (0.59)	1.7 (0.38)	11

Swiss Household Panel, wave 2019 and, SHP Covid-19 wave, weighted

## Footnotes

1 Based on authors’ calculations with data from the SHP Covid-19 study.

2 The data, questionnaires and user guide are accessible here: https://www.swissubase.ch/en/catalogue/studies/6097/16588/overview (accessed 11 February 2022).

3 See https://www.edk.ch/en/education-system/diagram?set_language=en (accessed 4 February 2022). Individuals who are currently enrolled in education are assigned the level of their education track.

4 Household Post-Government Income divided by the square root of the number of household members (OECD, 2008).

5 In a robustness test, we have assessed the relationship between being sometimes overwhelmed by homeschooling and wellbeing, while controlling for a number of other potentially relevant factors that may contribute to increasing parents’ subjective wellbeing. These factors include whether respondents’ are foreigners from a country where none of the Swiss languages is the official language, if there is more than one school-age child living in the household, respondents’ reported work-life balance prior to the pandemic, and survey mode in the pre-Covid-19-wave (i.e. telephone vs. web); we refer to this robustness check in Sect. 4.1..

6 Table 5 in the Appendix shows the results with standardized coefficients for continuous variables.

7 We have rerun the analysis for those respondents who were interviewed when homeschooling was effective. In both samples, tertiary educated individuals tend to be less overwhelmed by having to provide homeschooling.

8 Proxied by a birth year 2004–2014.

9 Based on the following question: “Since the introduction of the confinement measures, have there been any changes in how difficult it is to combine work and non-work life?” 1 = It has become much harder, …, 5 = It has become much easier.
